# Supplementary material for: General Practitioners’ Attitudes toward Municipal Initiatives to Improve Antibiotic Prescribing—A Mixed-Methods Study
Source: Antibiotics (Basel). 2019 Aug 17;8(3):120. doi: 10.3390/antibiotics8030120 (PMC6783816; doi:10.3390/antibiotics8030120)
Supplement: Supplementary file 1 [file antibiotics-08-00120-s001.pdf]

**E-mail with questions sent to general practitioners who consented to take part in the interview study (Part 1)**

Translated from Norwegian to English by SH.

“Thank you for participating in the study!

The questions we ask you to answer are given below. We are grateful if you can reply within a week. Feel free to give thorough and comprehensive answers if you have time; The more we know about your thoughts and attitudes around the questions, the more useful the results will be.

1. Most general practitioners find that they sometimes prescribe antibiotics without medical indication. Do you think this applies to you – and what are the main reasons for this?

2. The Government wants to reduce antibiotic use in the population by 30 per cent by the end of 2020.
a) What do you think of this goal?
b) Do you think you can reduce your own prescribing this much?
c) If yes: How? If no: why not?

3. One effective method of reducing antibiotic prescribing in general practice is to expose and discuss your own antibiotic prescription patterns with colleagues you are familiar with.
a) What do you think of such a method?
b) Would you participate in such a program if you were invited?
c) What do you think is needed to make such a program attractive to GPs?

4. According to the Regulations on the general practitioner scheme, the municipality shall ensure that systematic work is done on quality improvement in general practice. How would you like your municipal doctor to take measures (eg courses, group meetings) to improve the quality of the general practitioner's antibiotic prescribing?

Sincerely
Sigurd Høye
Postdoctoral Fellow
The Antibiotic Center for Primary Care
Department of General Practice
University of Oslo”
